# Integrated Clinical Genotype-Phenotype Characteristics of Blastic Plasmacytoid Dendritic Cell Neoplasm

**DOI:** 10.3390/cancers13235888

**Published:** 2021-11-23

**Authors:** C. Cameron Yin, Naveen Pemmaraju, M. James You, Shaoying Li, Jie Xu, Wei Wang, Zhenya Tang, Omar Alswailmi, Kapil N. Bhalla, Muzaffar H. Qazilbash, Marina Konopleva, Joseph D. Khoury

**Affiliations:** 1 Department of Hematopathology, The University of Texas, MD Anderson Cancer Center, Houston, TX 77030, USA; mjamesyou@mdanderson.org (M.J.Y.); sli6@mdanderson.org (S.L.); jxu9@mdanderson.org (J.X.); wwang13@mdanderson.org (W.W.); ztang@mdanderson.org (Z.T.); omar2016de@gmail.com (O.A.); 2 Department of Leukemia, The University of Texas, MD Anderson Cancer Center, Houston, TX 77030, USA; npemmaraju@mdanderson.org (N.P.); kbhalla@mdanderson.org (K.N.B.); mkonople@mdanderson.org (M.K.); 3 Department of Stem Cell Transplantation, The University of Texas, MD Anderson Cancer Center, Houston, TX 77030, USA; mqazilba@mdanderson.org

**Keywords:** blastic plasmacytoid dendritic cell neoplasm, NGS, DNA methylation pathway, overall survival

## Abstract

**Simple Summary:**

Mutation and protein-level profiling has expanded our understanding of the pathogenesis of blastic plasmacytoid dendritic cell neoplasm (BPDCN) and offered insights into potential therapeutic vulnerabilities. However, the prognostic impact of gene mutations in this disease remains controversial, and studies integrating the mutational landscape with cytogenetic and immunophenotypic characteristics in a real-world clinical context remain limited. We provide an overview of genotypic and phenotypic characteristics of a large, single-institution cohort of BPDCN patients. Besides gene mutations in DNA methylation and histone modification, BPDCN cells often harbor mutations affecting signal transduction, RNA splicing components, and transcription factors, particularly *ETV6* and *IKZF1*. Older age, multiple mutations, and mutations in the DNA methylation pathway are poor prognostic factors.

**Abstract:**

Blastic plasmacytoid dendritic cell neoplasm (BPDCN) is a rare, aggressive neoplasm derived from plasmacytoid dendritic cells. While advances in understanding the pathophysiology of the disease have been made, integrated systematic analyses of the spectrum of immunophenotypic and molecular alterations in real-world clinical cases remain limited. We performed mutation profiling of 50 BPDCN cases and assessed our findings in the context of disease immunophenotype, cytogenetics, and clinical characteristics. Patients included 42 men and 8 women, with a median age of 68 years (range, 14–84) at diagnosis. Forty-two (84%) patients had at least one mutation, and 23 (46%) patients had ≥3 mutations. The most common mutations involved *TET2* and *ASXL1*, detected in 28 (56%) and 23 (46%) patients, respectively. Co-existing *TET2* and *ASXL1* mutations were present in 17 (34%) patients. Other recurrent mutations included *ZRSR2* (16%), *ETV6* (13%), *DNMT3A* (10%), *NRAS* (10%), *IKZF1* (9%), *SRSF2* (9%), *IDH2* (8%), *JAK2* (6%), *KRAS* (4%), *NOTCH1* (4%), and *TP53* (4%). We also identified mutations that have not been reported previously, including *ETNK1, HNRNPK, HRAS, KDM6A, RAD21*, *SF3A1*, and *SH2B3.* All patients received chemotherapy, and 20 patients additionally received stem cell transplantation. With a median follow-up of 10.5 months (range, 1–71), 21 patients achieved complete remission, 4 had persistent disease, and 24 died. Patients younger than 65 years had longer overall survival compared to those who were ≥65 years (*p* = 0.0022). Patients who had ≥3 mutations or mutations in the DNA methylation pathway genes had shorter overall survival (*p* = 0.0119 and *p* = 0.0126, respectively). Stem cell transplantation significantly prolonged overall survival regardless of mutation status. In conclusion, the majority of patients with BPDCN have somatic mutations involving epigenetic regulators and RNA splicing factors, in addition to *ETV6* and *IKZF1*, which are also frequently mutated. Older age, multiple mutations, and mutations in the DNA methylation pathway are poor prognostic factors.

## 1. Introduction

Blastic plasmacytoid dendritic cell neoplasm (BPDCN) is a rare, aggressive hematologic malignancy derived from precursors of plasmacytoid dendritic cells. Most patients are older than 60 years at diagnosis, and men are affected more often than women. Patients usually present with widespread disease involving multiple anatomic sites, primarily the skin, bone marrow, peripheral blood, and lymph nodes. The diagnosis of BPDCN is based on a combination of morphologic features and characteristic immunophenotype. BPDCN cells are usually positive for CD4, CD56, CD123, TCL-1, and TCF-4 and negative for lineage-specific markers for B-cells, T-cells, and myeloid cells [1]. Despite initial response to chemotherapy, most patients develop early relapse with median overall survival of about one year [2].

Molecular profiling using next-generation sequencing (NGS) techniques has revealed recurrent gene mutations in BPDCN, expanding our understanding of its pathogenesis and providing novel therapeutic targets. Two genes that play a role in epigenetic regulation, *TET2* and *ASXL1*, have been identified as the most frequently mutated genes in BPDCN, and such mutations have been associated with worse clinical outcome in some studies [3]. However, the prognostic impact of gene mutations in this disease remains controversial, and studies integrating the mutational landscape with cytogenetic and immunophenotypic characteristics in a clinical, real-world context remain limited. In this study, we provide an integrated overview of the genotypic and phenotypic characteristics of a large, single-institution cohort of BPDCN patients.

## 2. Materials and Methods

### 2.1. Study Group

We identified all patients diagnosed with BPDCN seen at the University of Texas MD Anderson Cancer Center (MDACC) from 1 January 2000 to 31 December 2020. Among a total of 115 patients seen during this period, 50 were included in this study group on the basis of available mutation profiling data. All patients fulfilled the diagnostic criteria of BPDCN as specified in the World Health Organization classification [4]. Clinical and laboratory data were obtained by review of electronic medical records. This study was approved by the Institutional Review Board of MDACC and conducted in accordance with the Declaration of Helsinki.

### 2.2. Immunophenotypic Analysis

Immunophenotypic analysis using multicolor flow cytometry was performed on bone marrow (BM) aspirate specimens collected in EDTA anticoagulant tubes and processed within 12 hours of collection using a standard lyse/wash technique (PharmLyse^TM^, BD Biosciences, San Diego, CA, USA). For each analysis, a minimum of 200,000 events were acquired on FACSCanto II instruments (BD Biosciences). As described previously, the panel of monoclonal antibodies used included reagents specific for CD2, CD3 (surface and cytoplasmic), CD4, CD5, CD7, CD10, CD13, CD14, CD15, CD19, CD20, CD22, CD25, CD33, CD34, CD38, CD43, CD45, CD52, CD56, CD64, CD117, CD123, CD303, myeloperoxidase, HLA-DR, and TdT (BD Biosciences) [5]. An isotype control was used for each antibody.

Immunohistochemical studies were performed using formalin-fixed, paraffin-embedded tissue specimens using the avidin–biotin–peroxidase complex method on automated Leica Bond stainers (Leica Biosystems, Buffalo Grove, IL, USA). The antibodies utilized included TCL1, TCF4/CD123 multiplex stain, MYC, p53, and Ki-67 as described previously [6,7,8].

### 2.3. Conventional Cytogenetic and FISH Analysis

Conventional cytogenetic analysis was performed on metaphase cells prepared from bone marrow aspirates cultured for 24–48 hours without mitogens, using standard techniques. Giemsa-banded metaphases were analyzed, and the results were reported using the International System for Human Cytogenetic Nomenclature, 2016 (ISCN, 2016). FISH analysis was performed using probes specific for *CDKN2A* (p16), *ETV6*, *MYB, MYC*, or *TP53* on a subset of the cases with indications.

### 2.4. Next-Generation Sequencing

Genomic DNA extracted from BM was amplified and subjected to mutation analysis using a panel of 28 (*n* = 12 cases) or 81 (*n* = 32 cases) genes (commonly mutated in hematopoietic neoplasms) by NGS on an Illumina MiSeq platform (Illumina Inc., San Diego, CA, USA), as described previously [9].

### 2.5. Statistical Analysis

Statistical analysis was performed using GraphPad Prism 8 (GraphPad Software, San Diego, CA, USA). Overall survival was calculated from the date of initial diagnosis to the date of death or last follow-up. Survival was analyzed using the Kaplan-Meier method and was compared using the log-rank test. A value of *p* < 0.05 was considered statistically significant.

## 3. Results

### 3.1. Clinical Findings

Patients included 42 men and 8 women, with a median age of 68 years (range, 14–84 years) at diagnosis. All patients had bone marrow involvement, ranging from 5% to 95% (median, 30%). BPDCN involved skin and lymph nodes in 43 (86%) and 12 (24%) patients, respectively. Eight of 22 (36%) patients evaluated had tumor cells detected in cerebrospinal fluid. Five patients had another myeloid neoplasm subsequent to the diagnosis of BPDCN, including myelodysplastic syndrome (*n* = 3) and chronic myelomonocytic leukemia (*n* = 2). One patient from the latter group subsequently developed acute myelomonocytic leukemia.

Upon presentation at our hospital, 20 (40%) patients had leukopenia, and 5 (10%) had leukocytosis (median white blood cell count, 4.9 k/μL; range, 1.4–80.0 k/μL; reference range, 4–11 k/μL). Anemia was detected in 37 (74%) patients (median hemoglobin, 12.8 g/dL; range, 6.3–17.1 g/dL; reference ranges, 14–18 g/dL for men and 12–16 g/dL for women). Twenty-four (48%) patients had thrombocytopenia (median platelet count, 150 k/μL; range, 13–365 k/μL; reference range, 140–440 k/μL). Twenty-two (44%) patients had elevated serum lactate dehydrogenase (LDH) (median, 213 IU/L; range, 131–1800 IU/L; reference range, 135–225 IU/L), and 13 of 15 (87%) patients had elevated serum β2-microglobulin (β2M) level (median, 3.2 mg/L; range, 1.5–5.5 mg/L; reference range, 0.6–2.0 mg/L).

### 3.2. Immunophenotypic Findings

**Figure 1:**
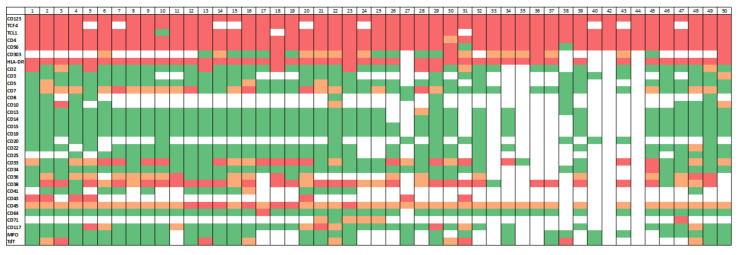
Immunophenotypic landscape of blastic plasmacytoid dendritic cell neoplasm.

The immunophenotypic characteristic of the study group are summarized in Figure 1.

Multiparameter flow cytometry immunophenotypic analysis demonstrated that the neoplastic cells were positive for CD4, CD45 (dim), CD123, and HLA-DR in all cases. Other markers expressed in subsets of patients included CD56 (47/49, 96%), CD38 (36/40, 90%), CD71 (4/5, 80%), CD36 (20/29, 69%), CD303 (16/26, 62%), CD33 (24/41, 59%), CD7 (21/44, 48%), TdT (8/28, 29%), CD117 (10/40, 25%), CD2 (9/42, 21%), CD10 (2/10, 20%), CD5 (3/38, 8%), CD41 (1/16, 6%), CD34 (2/39, 5%), cytoplasmic CD3 (1/22, 5%), CD13 (1/37, 3%), and CD64 (1/44, 2%). All cases assessed were negative for surface CD3 (*n* = 23), CD14 (*n* = 37), CD15 (*n* = 38), CD19 (*n* = 38), CD22 (*n* = 33), CD25 (*n* = 29), and myeloperoxidase (*n* = 28).

Immunophenotyping by immunohistochemistry further showed that BPDCN cells were positive for TCF4/CD123 (40/40, 100%), TCL1 (46/47, 98%), and CD43 (10/11, 91%) and negative for CD8 (*n* = 9) and CD20 (*n* = 18). The median Ki-67 proliferation index was 50% (range, 15% to 100%). Among the 27 patients evaluated for p53 expression, 1 patient (#28) expressed a mutant p53 pattern, and the finding correlated with the detection of *TP53*c.743G>A p.R248Q. Among the 24 patients evaluated by MYC immunohistochemistry, none showed protein overexpression.

### 3.3. Cytogenetic Findings

Conventional cytogenetic analysis was performed on 36 patients. Nineteen (53%) patients had diploid karyotype. Sixteen (44%) patients had a complex karyotype (≥3 abnormalities). One (3%) patient had two abnormalities (#12, -Y and +12). The most common aberrations were -13/del13q (*n* = 8), -5/del5q (*n* = 6), -7/del7q (*n* = 5), 12/del12p (*n* = 5), -11/del11q (*n* = 4), and -21 (*n* = 4) (Table 1).

FISH analysis was performed on a subset of cases. *ETV6* deletion and rearrangement were detected in three and two of seven cases assessed, respectively. *MYC* deletion and rearrangement were detected in one and one of six cases assessed, respectively. *TP53* deletion was detected in all five cases assessed. *CDKN2A* (p16) deletion was detected in one case assessed. One case showed a signal pattern suggestive of *MYB* gene rearrangement.

### 3.4. Gene Mutation Results

Mutations were detected in 42 (84%) patients: nine had one mutation, ten had two mutations, eight had three mutations, nine had four mutations, three had five mutations, two had six mutations, and one patient had eight mutations. In eight patients, no mutations were detected by 28-gene (*n* = 2) or 81-gene (*n* = 6) mutation profiling panels.

**Figure 2:**
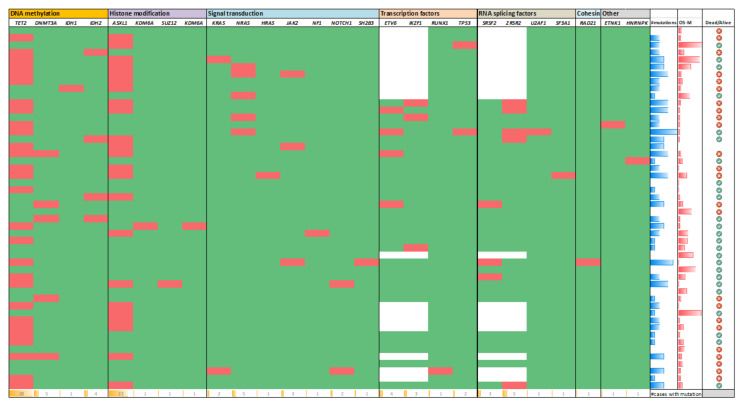
Somatic mutation landscape of blastic plasmacytoid dendritic cell neoplasm.

The most common mutations occurred in *TET2* and *ASXL1* genes, seen in 28 (56%) and 23 (46%) patients, respectively, and 17 (34%) patients had both *TET2* and *ASXL1* mutations. Mutations in *ASXL1* included 16 frameshift mutations and 7 nonsense mutations, with a median variant allele frequency (VAF) of 17.9% (range, 1.8% to 43.6%). Of note, *ASXL1* c.1934dupG p.E646fs was recurrently detected in 11 patients and *ASXL1* c.1990_1922del p.E635fs in 3 patients; other *ASXL1* mutations were non-recurrent. Mutations in *TET2* included 19 frameshift mutations, 16 missense mutations, 12 nonsense mutations, and 1 splice mutations, with a median VAF of 27% (range, 1.2% to 48.3%). The majority of *TET2* mutations were non-recurrent, except *TET2* c.4075C>T p.R1359C, which was detected in three patients. Half of the patients showed more than one *TET2* mutation, e.g., ten patients had two *TET2* clones, three patients had three *TET2* clones, and one patient had five *TET2* clones (Figure 2).

Other recurrently mutated genes included *ZRSR2* (5/32, 16%, median VAF 68%), *ETV6* (4/32, 13%, median VAF 17%), *DNMT3A* (5/50, 10%, median VAF 21.8%), *NRAS* (5/10, 10%, median VAF 21.4%), *IKZF1* (3/32, 9%, median VAF 46.3%), *SRSF2* (3/32, 9%, median VAF 14.3%), *IDH2* (4/50, 8%, median VAF 38%), *JAK2* (3/50, 6%, median VAF 14.3%), *KRAS* (2/50, 4%, median VAF 11%), *NOTCH1* (2/50, 4%, median VAF 8%), and *TP53* (2/50, 4%, median VAF 43.3%) (Figure 2).

Non-recurrent mutations included ETNK1, HNRNPK, HRAS, IDH1, KDM6A, NF1, RAD21, RUNX1, SF3A1, SH2B3, U2AF1, and SUZ12, seen in one patient each (Figure 2).

Sequential analysis performed on two patients showed *TET2* and *ASXL1* mutations initially, with one acquiring *IKZF1* and *ZRSR2* mutations five months later and the other acquiring *DNMT3A* and *ETV6* mutations two months later. In the first case, the patient had a normal diploid karyotype initially but subsequently had a complex karyotype, 46,XY,t(6;17)(q23;q22),der(7)add(7)(q11.2)t(?;21)(?;q11.2),add(21)(p12)[cp4]/47,XY,+mar[1]/46,XY[15].

### 3.5. Clinical Outcome

All patients received chemotherapy, 22 patients received tagraxofusp-based regimens and 18 patients received HCVAD-based regimens. Among all 50 patients, 36 received multi-agent chemotherapy, and 13 received tagraxofusp as a single agent. Twenty patients additionally received hematopoietic stem cell transplantation, 15 allogeneic and 5 autologous. With a median follow-up of 10.5 months (range, 1–71 months), 21 patients achieved complete remission, 4 patients had persistent disease, and 24 patients died. One patient was lost to follow-up.

**Figure 3:**
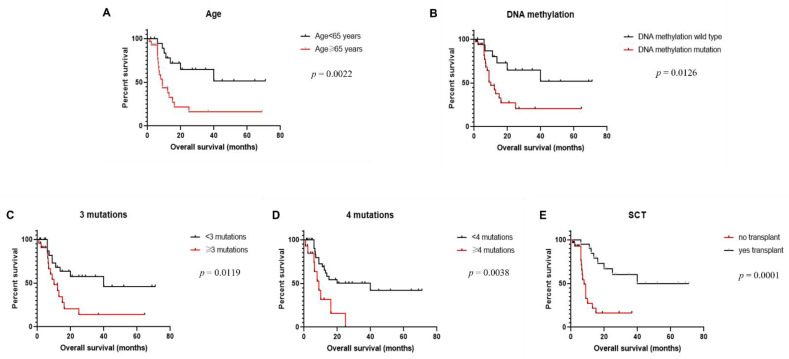
Overall survival of patients with blastic plasmacytoid dendritic cell neoplasm. (**A**) Comparison between patients ≥65 years and <65 years of age. (**B**) Comparison between patients with and without mutations affecting DNA methylation pathway. (**C**) Comparison between patients with <3 vs. ≥3 mutations. (**D**) Comparison between patients with <4 vs. ≥4 mutations. (**E**) Comparison between patients who did and those who did not receive hematopoietic stem cell transplantation (SCT).

We performed univariate analysis to evaluate the association between clinical, laboratory, cytogenetic, and mutation data and overall survival (Table 2). Age older than 65 years (*p* = 0.0022) was associated with shorter overall survival (Figure 3A). We did not observe any significant difference between overall survival and mutation status of each individual gene assessed, including *TET2*, *ASXL1*, *DNMT3A*, *NRAS*, and *ZRSR2*. No difference in survival was detected among patients with no mutation or 1–2 mutations. We further grouped genes that were mutated in our study based on their function, and we analyzed the correlation between survival and mutation status based on functional pathways. We identified significantly reduced overall survival among patients with gene mutations involved in the DNA methylation pathway, including *TET2*, *DNMT3A*, *IDH1*, and *IDH2*, as compared to patients with a wild-type genotype for these genes (*p* = 0.0126) (Figure 3B). No statistically significant difference was detected between overall survival and gene mutations involved in histone modification (*ASXL1*, *KDM6A*, and *SUZ12*), signal transduction pathway (*KRAS*, *NRAS*, *HRAS*, *JAK2*, *NF1*, *NOTCH1*, and *SH2B3*), transcription factors (*ETV6, TP53*, *IKZF1*, and *RUNX1*), or RNA splicing factors (*SF3A1*, *SRSF2*, *ZRSR2*, and *U2AF1*). Moreover, we found that patients with three or more mutations had significantly shorter overall survival than those with two or fewer mutations (*p* = 0.0019), and the significance was more pronounced when we used four or more mutations as the cutoff (*p* = 0.0038) (Figure 3C,D). Finally, patients who received hematopoietic stem cell transplantation had significantly longer overall survival compared to those without transplantation (*p* = 0.0001) (Figure 3E), and such a difference was not dependent on mutation status. There was no significant association between overall survival and other parameters assessed, including sex, white blood cell count, hemoglobin, platelet count, level of LDH, level of β2M, and presence of cytogenetic aberrations.

## 4. Discussion

Collectively, gene mutations in BPDCN affect several functional classes of genes, mainly DNA methylation, histone modification, signal transduction, transcription factors, cell-cycle regulation, and splicing factors [10,11,12,13,14]. However, the reported frequency of each mutation has varied widely, and the impact of these mutations on prognosis has remained incompletely understood due to the rarity of this disease. In this study, we assessed the mutation profile and its clinical impact in 50 BPDCN cases evaluated by NGS using targeted panels that sequence genes commonly mutated in hematologic neoplasms. We detected mutations in the interrogated genes in 84% of the cases, and 66% of patients had more than one mutation. Genes mutated in our study included those affecting DNA methylation (*TET2*, *DNMT3A*, *IDH1*, and *IDH2*), histone modification (*ASXL1, KDM6A*, and *SUZ12*), signal transduction (*NRAS, KRAS, HRAS, JAK2, NF1, NOTCH1, SH2B3*), transcription factors (*ETV6, TP53, IKZF1, RUNX1*), RNA splicing factors (*SF3A1*, *SRSF2, ZRSR2, U2AF1*), and cohesin complex component (*RAD21*). In keeping with previous studies, our results showed that *TET2* and *ASXL1* were the most frequently mutated genes, seen in 56% and 46% of cases, respectively. All *ASXL1* mutations were frameshift or nonsense, and most were located within or upstream of the catalytic domain, which may cause disruption of protein function [15]. Similarly, *TET2* mutations were scattered along all exons, and most (69%) were frameshift or nonsense with a potential to affect the function of the protein [16]. In two cases for which we had sequential samples, both had *TET2* and *ASXL1* initially, and they subsequently acquired additional mutations that included *IKZF1* and *ZRSR2* in one and *DNMT3A* and *ETV6* in another. These findings suggest that *TET2* and *ASXL1* mutations may represent early events in the pathogenesis of BPDCN, whereas the other mutations are acquired secondarily due to genomic instability and clonal evolution resulting from epigenetic dysregulation. In addition, we identified a few mutations that have not been reported previously in BPDCN, including *ETNK1, HNRNPK, HRAS, KDM6A, RAD21*, *SF3A1,* and *SH2B3*, or have only been described in single case reports, including *NF1*, *NOTCH1*, and *SUZ12* [12,17,18]. Similar to *ASXL1* and *TET2* mutations, most of these mutations, such as *ZRSR2* (3/5), *DNMT3A* (3/7), *IKZF1* (2/3), *ETV6* (2/4), *NOTCH1* (1/2), *KDM6A* (1/1), and *SH2B3* (1/1) were also frameshift, premature stop, or splice site mutation, with probable loss of function of the encoded proteins. By contrast, *TP53* mutations are uncommon in BPDCN even though copy loss at the *TP53* locus is not infrequent. Surprisingly, genes that are most frequently mutated in AML, in particular *FLT3* and *NPM1*, as well as some mutated genes reported previously, such as *EZH2*, *KIT*, and *SF3B1*, were not mutated in our study group [11,13].

*ASXL1* encodes a polycomb repressive complex protein with a role in chromatin regulation. *ASXL1* interacts with polycomb complex proteins and other transcription activators or repressors to regulate transcription and translation. Recurrent *ASXL1* mutations have been reported in a variety of myeloid neoplasms and have been linked to an aggressive disease course, blast transformation, and poor clinical outcome [19,20,21]. *TET2* plays important roles in stem cell self-renewal, lineage commitment, and terminal differentiation of monocytes [19,20,21]. Loss-of-function mutations of *TET2* are common in both myeloid and lymphoid neoplasms and are associated with DNA hypermethylation and abnormal gene expression in hematopoietic cells [22]. Its role in prognosis has been controversial. While *TET2* mutations have been associated with blast transformation in myeloproliferative neoplasms and unfavorable prognosis in acute myeloid leukemia [23,24,25], other studies failed to identify a strong association between *TET2* mutations and overall survival [20,26]. We did not detect any correlation between overall survival and mutation status of each individual gene assessed. However, upon grouping of the genes based on their functions, patients with gene mutations in the DNA methylation pathway had significantly reduced overall survival. There was no difference between survival and mutations affecting other functional classes, including histone modification, signal transduction, transcription factors, and RNA splicing. Further analysis demonstrated that patients who carried three or more mutations had significantly shorter overall survival compared to those with two or fewer mutations, and patients with four or more mutations had even inferior clinical outcome. Therefore, we observed a likely synergistic effect of mutations, either based on the function pathway affected or based on the number of mutations, on the clinical outcome, which may be explained by the more complex genetic landscape and more implicated signaling pathways in patients with multiple mutations.

Our results show that genes encoding RNA splicing components are frequently mutated in BPDCN, with a collective frequency of 23%. Renosi et al. [27] performed NGS targeting 68 genes on 13 cases of BPDCN and detected *ZRSR2* mutations in 4 (31%) cases and *SRSF2* mutations in 2 (15%) cases. Summerer et al. [28] analyzed 1367 mutations in 1210 genes in 21 BPDCN cases and found mutations in *SRSF2* in 7 (33%), *SF3B1* in 2 (10%), *U2AF1* in 2 (10%), and *ZRSR2* in 2 (10%) cases. Loss-of-function mutations in *ZRSR2* have been shown to impair plasmacytoid dendritic cell activation and apoptosis after inflammatory stimuli, thus may impair immunity and predispose to leukemic transformation [29]. The presence of frequent mutations of RNA splicing factor may result in impairment of RNA splicing and defective assembly of gene transcripts, which could contribute, at least in part, to the disruption of signaling pathways involved in plasmacytoid dendritic cell maturation and thus BPDCN tumorigenesis.

Another recently discovered recurrent mutation in BPDCN is *IKZF1*, a member of the IKAROS gene family. *IKZF1* encodes a transcription factor that is essential for B-cell development and regulates the differentiation and function of plasmacytoid dendritic cells [30]. IKZF1 deficiency has been correlated with a reduced number of plasmacytoid dendritic cells and decreased production of IFN-alpha, TNF, and IL-12 in a mouse model [30]. Menezes et al. [11] first reported IKAROS family gene mutation (*IKZF1*, *IKZF2,* and *IKZF3)* in 5 of 25 (20%) of BPDCN cases. Torres et al. [31] performed whole-genome sequencing and RNA sequencing, which revealed that *IKZF1* was focally inactivated via recurrent structural alterations. IKZF1 deficiency leads to up-regulation of cellular processes responsible for cell–cell and cell–extracellular matrix interaction and may play a central role in the pathobiology of BPDCN.

Patients with BPDCN carry heterogeneous karyotypic aberrations. It has been reported that approximately 60% of patients have a complex karyotype, with imbalanced chromosomal losses being most common, including -5/del(5q31) (*NR3C1*), del(6q25) (*SYNE1*), -7/del(7q), del(7p12.2) (*IKZF1*), del(9p21) (*CDKN2A, CDKN2B*), del(11q), del(12p13) (*CDKN1B, ETV6*), del(13q13-q14) (*RB1*), del(15q), and del(17p13) (*TP53)* [27,32]. These changes may lead to loss of tumor suppressor genes and contribute to tumorigenesis. It has been reported that biallelic loss of 9p21.3 or mono-allelic loss of 5q31 conferred poor clinical outcome [33,34]. However, most other cytogenetic aberrations carry no prognostic value. The most common aberrations were similar to those reported, including -13/del(13q), -5/del(5q), -7/del(7q), -12/del(12p), and -11/del(11q). We identified a high rate of 12p13/*ETV6* deletions/rearrangements (5/7, 71%) in BPDCN. Genomic aberrations involving 12p13 locus containing *ETV6* have been reported as the most common findings in BPDCN and may represent early clonal events [35]. Similar to other published data, 8q24/*MYC* abnormalities were detected in one-third of the patients, and one patient had t(6;8)(p21;q24). Rearrangements involving the *MYC* locus at 8q24 may lead to MYC protein overexpression and correlate with inferior clinical outcome [36]. An extra copy of the *MYB* gene or *MYB* rearrangement at 6q23.3 was detected in one patient who was a 78-year-old man, in contrast to prior studies that showed that *MYB* rearrangement was more common in children and young adults [37]. The rearrangement has been shown to induce *MYB* activation by truncating the negative regulator domain [37].

A variety of chemotherapeutic regimens designed for myeloid and lymphoid neoplasms have been used to treat patients with BPDCN. However, most patients experience an aggressive clinical course with poor outcomes despite intensive chemotherapy. Identification of recurrent gene mutations and rearrangements may aid in the development of novel therapeutic approaches. Sapienza et al. [14] performed whole-exome sequencing and RNA sequencing on 14 BPDCN cases, identified deregulation of the epigenetic program as a genetic hallmark of BPDCN, and suggested a novel therapeutic approach based on the combination of two hypomethylating agents, 5′-azicytidine and decitabine. Stem cell transplantation has been shown to result in durable remissions [38,39]. Our studies also showed that stem cell transplantation significantly prolonged the overall survival of patients with BPDCN.

## 5. Conclusions

In summary, we show that besides gene mutations in DNA methylation and histone modification, mutations affecting signal transduction, RNA splicing factors, and transcription factors, particularly *ETV6* and *IKZF1*, are frequently present in BPDCN. We identified that older age, mutation in genes involved in DNA methylation, and presence of multiple (≥3) mutations were poor prognostic markers in this disease.

## Figures and Tables

**Table 1:** Karyotypes of cases of blastic plasmacytoid dendritic cell neoplasm.

Case	Karyotype
1.	46,XY[19]/47,XY,+1[1]
2.	46,XY,t(1;6)(p21;p36.3),del(5)(q13q33),der(7)t(1;7)(q12;p22),del(11)(q13q23),del(12)(p11.2p13),add(15)(q15)[10]
3.	42,XY,-5,-12,-15,add(17)(p11.2),add(17)(p13),-18,add(20)(q13.3)[3]/43,idem,+mar[8]/42,idem,-13,+mar[2]/46,XY[4]
4.	46,XY,+1,add(1)(p13),der(1)dup(1)(q21q32)add(1)(q42),-2,-4,+11,add(12)(p11.2), -13,-21,+2mar[8]/48,XY,+1,der(1)dup(1)(q21q32)add(1)(q42),-2,-4,+11,add(12)(p11.2),-13,+3mar[1]/46,XY[11]
5.	46,XY[20]
6.	46,XY[20]
7.	46,XY,t(2;7)(p23;p15),add(9)(p13),del(9)(p21)[20]
8.	46,XX[20]
9.	46, XY[20]
10.	45,XY,der(6)del(6)(q13q22)t(6;9)(q25;q34),-7,der(9)del(9)(p21)t(6;9),der(9;15)(q10;q10),-13,-14,+2~3mar[cp6]/46,XY[14]
11.	46,XY,t(6;17)(q23;q22),der(7)add(7)(q11.2)t(?;21)(?;q11.2),add(21)(p12)[cp4]/47,XY,+mar[1]/46,XY[15]
12.	46,X,-Y,+12[3]/46,XY[17]
13.	44~45,XX,del(5)(q33q35),del(6)(q13q23),der(7;9)(q10;q10),-13[cp12]/46,XX[8]
14.	44,XX,add(1)(p36.1),del(9)(p21),add(12)(p12),-13,add(17)(p12),-21,-22,add(22)(q12),+mar[5]/46,XX[15]
16.	48,XY,t(6;8)(p12;q24.2),add(7)(p11.2),del(13)(q12q22),+16,add(20)(q11.2),+21[10]/46,XY[10]
17.	46, XY[20]
18.	46,XY[20]
19.	86~87,XXYY,-5,add(6)(q21)x2,-11,add(11)(q23),-12,i(12)(q10),add(19)(p13.3),+3mar[cp3]/46,XY[17]
20.	46,XY[20]
21.	44~47,XY,add(3)(p13),add(5)(p15.1),del(5)(q13q33),-8,add(8)(q13),del(9)(p21),del(9)(q13q22), del(10)(q24),del(11)(q13q23),del(12)(p12),- 13,del(13)(q12q22),+del(17)(p11.2),add(20)(q13.3),add(21)(p13),+mar[cp11]/46,XY[9]
22.	42,XY,-6,-7,-8,add(9)(q13),der(11)add(11)(p15)add(11)(q24),der(13;15)(q10;q10),add(17)(p11.2),-22,+2mar[4]/46,XY[16]
23.	46~48,XX,del(6)(q13q23),+der(7)add(7)(p13)del(7)(q11.2q22)add(7)(q32)x2,der(7)add(7)(p13)del(7)(q11.2q22), add(7)(q32)x2,-8,add(9)(p24),add(12)(p13),-14,del(16)(q23),+21,+22,+mar[cp19]/46,XX[1]
24.	46,XY,del(12)(p11.2p13)[1]/46,XY[19]
25.	46,XY,del(3)(p11.2)[1]/46,XY[19]
26.	47,XY,+Y[1]/46,XY[19]
27.	46,XY,inv(9)(p12q13)[20]
28.	45,XY,?del(6)(q24),-7,?i(7)(q10),+?12,-14,15,+mar[3]/46,XY[12]
29.	46,XY,del(12)(q14)[1]/46,XY[19]
30.	46,XX[20]
31.	46,XX[20]
32.	46,XY[20]
33.	46,XY[19]
46.	46,XY,t(3;9)(q25;q34)[1]/46,XY[19]
47.	45,XY,i(7)(q10),i(14)(q10),-21[2]/46,XY[18]
48.	46,XY[4]
49.	81~82,XXYY,+1,+1,add(1)(p22),del(1)(p31),del(1)(q21),add(2)(p25),add(2)(q37),-3,-4,-5,-5,-6,-9,-10,-12,-13,-15,+16,+16,-17,-18,-20,-21,+2~4mar[cp2]/46,XY[18]

**Table 2:** Univariate analysis of clinical, laboratory, and molecular genetic data to predict overall survival.

Features	*p* Value	HR Ratio	95% CI of HR
Age (<65 years vs. ≥65 years)	0.0022 *	0.287	0.1273–0.6473
Sex (men vs. women)	0.8444	1.112	0.3941–3.136
WBC (low vs. normal vs. high)	0.0561	NA	NA
Hb (low vs. normal)	0.6007	1.293	0.5159–3.243
Platelet (low vs. normal)	0.4	1.403	0.6285–3.133
LDH (high vs. normal)	0.6043	1.24	0.5522–2.784
β2M (high vs. normal)	0.7633	0.8077	0.2143–3.044
Karyotype (abnormal vs. diploid)	0.0969	0.516	0.2094–1.272
Mutation (yes vs. no)	0.2902	0.5724	0.2296–1.427
*ASXL1* (mut vs. wt)	0.2618	0.6382	0.2845–1.432
*DNMT3A* (mut vs. wt)	0.1098	0.4403	0.1016–1.909
*NRAS* (mut vs. wt)	0.8036	1.198	0.3122–4.595
*TET2* (mut vs. wt)	0.0743	0.4878	0.2182–1.090
*ZRSR2* (mut vs. wt)	0.3374	0.4977	0.0692–3.581
DNA methylation (mut vs. wt)	0.0126*	0.3399	0.1527–0.7567
Histone modification (mut vs. wt)	0.2697	0.6429	0.2867–1.442
Signal transduction (mut vs. wt)	0.4435	1.505	0.5825–3.886
Transcription factors (mut vs. wt)	0.2226	0.5627	0.1843–1.718
RNA splicing factors (mut vs. wt)	0.5414	0.7159	0.2080–2.464
#mutation (<3 vs. ≥3)	0.0119 *	0.3766	0.1624–0.8737
#mutation (<4 vs. ≥4)	0.0038 *	0.3246	0.1099–0.9589
Transplantation (yes vs. no)	0.0001 *	0.2378	0.1010–0.5597

β2M, β2-microglobulin; Hb, hemoglobin; LDH, lactate dehydrogenase; mut, mutated; NA, not available; WBC, white blood cell count; wt, wild type, * Denotes *p* Values that are statistically significant.

## Data Availability

The data presented in this study are available upon request from the corresponding authors.
